# A Scoping Review of the Social Determinants of Pediatric and Adolescent Obesity

**DOI:** 10.1155/ijpe/8871022

**Published:** 2025-04-28

**Authors:** Deepali K. Ernest, Elizabeth A. Onugha, Bipin Singh, Shreela V. Sharma, Jayna M. Dave

**Affiliations:** ^1^Department of Epidemiology, University of Texas Health Science Center at Houston School of Public Health, Houston, Texas, USA; ^2^Center for Health Equity, The University of Texas Health Science Center at Houston School of Public Health, Houston, Texas, USA; ^3^Department of Pediatrics, Baylor College of Medicine, Houston, Texas, USA; ^4^Texas Children's Hospital, Houston, Texas, USA; ^5^USDA/ARS Children's Nutrition Research Center, Houston, Texas, USA

## Abstract

**Context:** Social determinants of health (SDOH) play a large role in pediatric and adolescent metabolic health worldwide.

**Objective:** The study is aimed at exploring key SDOH related to childhood obesity, worldwide.

**Methods:** Primary research articles from PubMed, Embase, and Medline databases published between 2013 and 2023, had a study population of 0–19-year-olds, and examined the association between WHO-defined SDOH and childhood obesity were included. Non-English papers and those outside WHO-defined SDOHs were excluded. Two reviewers independently performed a blinded screening of titles and abstracts, followed by a full-text assessment of selected articles.

**Results:** Of the 703 initial articles, 22 duplicates were excluded, leaving 681 unique articles from PubMed (*N* = 274), Medline (*N* = 43), and Embase (*N* = 364). Initial screening excluded 579 articles, and full-text screening excluded 61 more, resulting in 41 final articles. Reasons for exclusion primarily involve missing SDOH exposure or weight-related outcomes and articles being reviews, editorial/opinion pieces, or interventional studies. Most included studies were cross-sectional (*N* = 25) and conducted in North America (*N* = 22). The average study sample size was 43,640 participants. These studies focus on socioeconomic determinants, neighborhood characteristics, food environment, healthcare access, educational determinants, and immigration-related factors. Obesity-related outcomes included general obesity, severe obesity, abdominal obesity, weight gain, BMI/weight categories, and continuous BMI measures.

**Conclusion:** Key SDOHs of childhood obesity include socioeconomic status, neighborhood characteristics, food environment, healthcare access, immigration, and culture. Despite diverse regional studies, there is a notable gap in US-specific data on SDOH of childhood obesity, especially by race and ethnicity. Further research is needed to better understand these determinants and their impact on pediatric metabolic health.

**Summary**

This review explores key social determinants of childhood obesity globally, identifying socioeconomic status, neighborhood, food environment, healthcare access, and cultural factors as major contributors.

## 1. Introduction

Human health and well-being extend beyond biological factors; they encompass a broad spectrum of social elements that significantly shape an individual's life. Social determinants of health (SDOH) are nonmedical factors, like nativity, education, income, food insecurity, housing, and access to healthcare, that significantly impact health outcomes [1]. These factors that interact with socioeconomic status (SES), cultural influences, and environmental conditions account for a substantial portion of global health disparities, in both acute and chronic health [1].

SDOH are well-established drivers of metabolic health among adults, especially in the context of obesity [2, 3]. Research in the United States has linked obesity rates to geographical region, age, race/ethnicity, income, and education [4–8]. For instance, states like Colorado with a relatively higher median income level reported lower rates of obesity compared to states like Mississippi, which had lower median income levels (obesity prevalence: 24.2% vs. 39.7%) [3, 9]. Additionally, evidence suggests that minority racial and ethnic groups are overrepresented in low-income neighborhoods [10], which have a higher likelihood of environmental exposures contributing to obesity [11]. The intersection of multiple determinants of obesity such as neighborhood, income level, and race/ethnicity suggests that they may independently and jointly affect the prevalence of obesity. For instance, US adults with lower income and education levels, who are older, and of African American/Black descent have higher rates of obesity compared to those with higher income or education levels and of other races/ethnicities [3]. Similar trends have been observed globally, further solidifying the worldwide impact of SDOH on adult obesity [12–15].

Given the alarming rise in childhood and adolescent obesity globally [16], it is critical to understand the influence of SDOH on this younger, more vulnerable population [8, 9]. While school-based programs can improve metabolic health [17], addressing obesity requires a multipronged approach, especially in the socioenvironmental front [9]. Understanding and addressing SDOH that influence childhood obesity is essential for developing effective interventions and policies, ultimately fostering healthier and more resilient communities.

Numerous studies have examined the association between SDOH and pediatric/adolescent obesity in individual populations [18–22]. However, a comprehensive understanding of how various SDOH domains [23] like socioeconomic, environmental, cultural, contextual, and behavioral domains influence pediatric and adolescent obesity is lacking. This scoping review is aimed at addressing this gap by (1) consolidating global literature on SDOH associated with pediatric and adolescent obesity and (2) comprehensively identifying key socioeconomic, environmental, cultural, and behavioral factors that influence obesity among children and adolescents worldwide.

## 2. Methods

### 2.1. Search Strategies

We performed a comprehensive literature search using electronic databases, including PubMed, Embase, and Medline. The search terms included keywords extracted from the World Health Organization SDOH domains such as income and social protection, education, unemployment, job insecurity, working life conditions, food insecurity, housing, basic amenities, environment, early childhood development, social inclusion and nondiscrimination, structural conflict, and access to affordable health services of decent quality [1]. We also included terms related to childhood or adolescent obesity such as SDOH, health social determinants, structural determinants of health, health structural determinants, obesity, overweight, body mass index (BMI), pediatric, childhood, child, children, adolescent, young adult, and teenager.

### 2.2. Inclusion and Exclusion Criteria

We limited the search to primary research articles with one or more of the exposures falling within SDOH domains, while either the primary or secondary outcome had to be weight-related (i.e., weight categories, waist-to-hip ratio, obesity scoring system, or BMI). Participants included in the studies had to be between 0 and 19 years old and without major health conditions. We only considered articles published between 2013 and 2023. We excluded articles that were unavailable in the English language, inaccessible, intervention studies, editorials, reports, or reviews. No geographic, sex/gender, or race-specific restrictions were applied.

### 2.3. Article Selection

**Figure 1:**
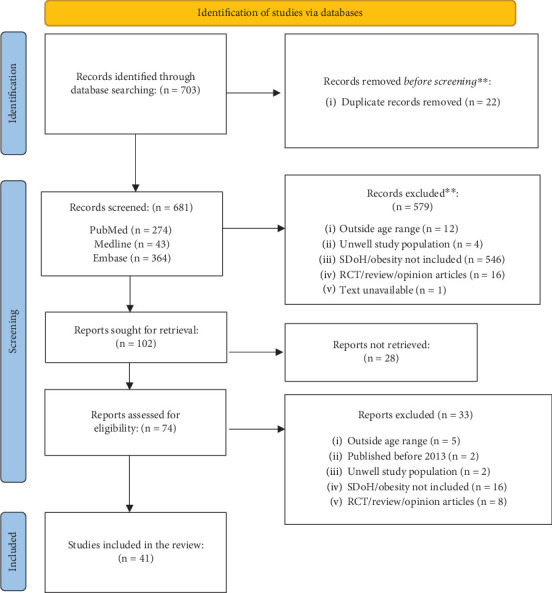
Inclusion and exclusion process of articles collected from PubMed, Medline, and Embase (*N* = 703).

Two independent reviewers selected articles through an iterative process to ensure objectivity. The selection process involved three rounds of screening: (1) blinded screening of title and abstracts for initial assessment of relevance and compliance with inclusion criteria, (2) full-text assessment for detailed evaluation of articles for eligibility, and (3) rescreening of excluded articles to ensure no eligible articles were missed. We documented the reason for exclusion. Any article that received conflicting decisions from reviewers was jointly reviewed and resolved with consensus and discussion. We identified and excluded duplicate articles before screening. The article selection process is outlined in detail in Figure 1.

### 2.4. Data Extraction and Analysis

We extracted data from the selected articles and organized it using Google Sheets. The information collected included study title, author list, keywords, publication year, study design, and geographic location. Additional details included study population, age group, sample size, SDOH domain, outcome, data collection method, statistical methods, main findings, and strengths/limitations of the study. Article characteristics are consolidated and summarized in Table 1. Study components such as design, data collection, statistical methodology, and main findings are summarized in Table 2.

## 3. Results

### 3.1. Study Inclusion

The initial article search yielded a total of 703 articles. After excluding 22 duplicates, we were left with a total of 681 articles from PubMed (*N* = 274), Medline (*N* = 43), and Embase (*N* = 364) (Figure 1). The initial blinded screening of titles and abstracts yielded 579 articles that did not fit the inclusion criteria—these articles were excluded from the review. The subsequent full-text review of the remaining 102 articles resulted in the exclusion of 61 more articles. Lastly, a rescreening of the 638 excluded articles found no additional eligible articles. As a result, we included a total of 41 articles in this review.

During the blinded screening process, missing SDOH exposure or inappropriate weight-related outcome was the most common reason for exclusion (*N* = 546). Other reasons included study design (i.e., intervention studies, review articles, editorials, and opinion pieces [*N* = 16]), participants not meeting the 0–19-year age criteria (*N* = 12), pre-existing medical conditions (*N* = 4), and abstract unavailability (*N* = 1). During the full-text review, 61 articles were excluded for several reasons. The most common was the unavailability of the full text (*N* = 28). Other reasons included non-SDOH exposures, outcomes unrelated to obesity (*N* = 16), and excluded study design (*N* = 8). Some studies were outside the 0–19-year age range (*N* = 5), involved pre-existing medical conditions (*N* = 2), or had a publication year preceding 2013 (*N* = 2).

### 3.2. Characteristics of Included Studies

General article metrics indicated that many of the included studies were of cross-sectional design (*N* = 25) and published in 2022 or 2023 (*N* = 14). Most studies were conducted in North America (*N* = 21) across Canada, United States, and Mexico, followed with seven studies in Europe (Italy, Norway, and the United Kingdom). The Middle East had five studies (Iran and United Arab Emirates), and South America had four (Colombia, Argentina, Chile, and Brazil). Asia contributed two studies (Korea and Indonesia), while Africa (Ghana) and Australia each had one. The studies had a mean sample size of 43,640 participants, with the smallest study having 102 participants and the largest having 818,210 participants [45, 47].

Of the 41 included articles, 26 explored socioeconomic determinants of childhood obesity. These included socioeconomic inequalities, material or social deprivation, parental employment, income level, poverty status, and attending schools in socioeconomically disadvantaged areas. Nine articles examined neighborhood characteristics such as walkability scores, presence of sidewalks, parks or playgrounds, recreational centers, community centers, geographical location (urban vs. rural), and exposure to environmental pollutants. Three articles investigated food environment factors such as food insecurity and receiving food assistance. One article focused on healthcare access/utilization through insurance coverage and general insurance type (private vs. public). Additionally, two other articles examined immigration-related factors such as immigrant status and connections to the migrant social network. It is important to note that many articles examined more than one SDOH from different domains.

A variety of obesity-related outcomes were studied across the 41 included studies, including general obesity (*N* = 27), abdominal obesity (*N* = 1), severe obesity (*N* = 1) (BMI ≥ 40), weight gain (*N* = 1), BMI weight categories (*N* = 5), and continuous BMI measures (*N* = 6).

### 3.3. Review Findings

This review identified a total of 41 primary research articles published between 2013 and 2023 that studied the association of SDOH with pediatric and adolescent obesity. Nearly 76% of these studies (*N* = 31) focused on populations from high-income countries such as the United States (*N* = 15), Canada (*N* = 5), United Kingdom (*N* = 3), Norway (*N* = 2), Italy (*N* = 2), Australia (*N* = 1), United Arab Emirates (*N* = 1), South Korea (*N* = 1), and Chile (*N* = 1). The remaining 10 studies focused on populations from Argentina (*N* = 1), Brazil (*N* = 1), Colombia (*N* = 1), Iran (*N* = 4), Mexico (*N* = 1), Ghana (*N* = 1), and Indonesia (*N* = 1). Most of these studies highlighted significant associations between childhood obesity and SES, neighborhood characteristics, education, food environment, healthcare access/utilization, and immigration-related factors.

#### 3.3.1. SES

SES is influenced by factors such as family income, parental education, employment status, and family affluence [62]. Many articles included in this review demonstrated significant associations between SES and childhood obesity. A Canadian cross-sectional study of Aboriginal (Metis) children (6–14 years) found an elevated risk of obesity among children living in single-parent households, with a primary caretaker who had less than a high-school education and an annual household income below $63,000, compared to their peers [30]. In contrast, cross-sectional studies from Azerbaijan and Iran suggest a higher likelihood of obesity with an increase in annual household income level and a reduction in poverty [43, 44]. The SES of children's residential areas also impacted their risk of obesity [63]. Studies from Iran and Colombia indicate a higher likelihood of overweight (OR = 2.36; 95% CI: 1.37, 4.08) or obesity (OR = 3.25; 95% CI: 1.89, 5.57) among children (6–19 years) living in high SES areas compared to those in low SES areas [43, 48]. Furthermore, children (13–19 years) who attend public schools are 67% more likely to be overweight or have obesity compared to those attend private schools [31]. A cross-sectional study from Ghana, Africa, also noted higher prevalences of overweight and obesity among children (0–5 years) in the fourth wealth index quintile compared to those in the poorest quintile (PR = 1.01; 95% CI: 1.01, 1.02) [41].

Parental education attainment, employment status, and household features (i.e., single vs. dual parent household, single vs. multiple sibling family, household income, parent vs. grandparent caregiver) also impacts the risk and likelihood of childhood obesity [60]. Multiple European studies, including the UK-based Millennium Cohort Study, have observed a 60% increase in the likelihood of obesity (OR = 1.60; 95% CI: 1.4, 1.8) among children (11 years) whose mothers had no educational qualification [39]. These observations indicate a significant widening of the absolute and relative inequalities in childhood obesity by maternal education attainment over time [39, 52, 54, 60].

Among young Norwegian children (6 years), a significant progression towards obesity was noted with low parental education, paternal unemployment, non-Western ethnicity parents, single-parent households, and being an only child [42]. Researchers in Italy evaluated the impact of parental education and SES disparities on obesity. They concluded that 43% of Apulian children (8–9 years) in Italy with obesity, whose parents had low levels of education and severe economic difficulties, could reduce their weight if social inequalities were addressed [46]. Additionally, SES-related SDOH affecting childhood obesity included being looked after by a grandparent after school, only primary-level maternal education, and material deprivation [20, 51]. Overall, findings regarding SES align with other studies from the Middle East (Iran) [40], Europe (Norway) [55], Asia (Korea) [47], and North/South America (United States, Canada, Brazil) [30, 52, 56, 59–61]. One specific study noted the effect measure modification of the relationship between parental education and childhood obesity by race and ethnicity [50]. Educational disparities affect dietary knowledge, food purchasing behavior, and perceptions of nutritious food items [64], with education being the socioeconomic indicator most significantly predicting diet quality [65].

On the contrary, a few studies found either a null or protective effect of SES factors on childhood obesity. For instance, studies from Indonesia and Argentina found no significant effects of family affluence, parental education, single-parent households, and income on childhood obesity [38, 45]. Additionally, studies from Europe, Australia, and the United States found a higher likelihood of obesity among both males and females in the low SES group, compared to those in middle and/or high SES groups [52, 54, 60]. This contrasts with the findings from previously discussed studies [49, 53, 54, 57, 58]. While low SES is associated with a higher prevalence of obesity in developed countries, a positive SES–obesity relationship is observed in developing nations.

The contrasting effect of SES on childhood obesity has been examined by previous studies and termed “the obesity–poverty paradox” [66]. Economic globalization and income growth in developing countries bring an influx of westernized services, including food delivery and easy access to calorie-dense, processed foods that lack nutritional value, contributing to the rise of obesity [66]. In developing countries, the purchase and consumption of food from commercial establishments is considered a luxury and generally affordable to populations with high SES [67]. This shift towards westernized food markets and change in food behaviors may contribute to the observed association between high SES and obesity in developing countries like India and Ghana [68].

On the other hand, obesity tends to be associated with low SES among developed countries in North America and Europe [69]. This can be attributed to the lack of access to fresh food and the high cost of purchasing healthy and organic food. Low-income households often rely on the cheapest food options available to them, which tend to be highly processed foods with empty calories [69]. Additionally, the lack of physical activity due to the high cost of sports equipment/facilities and limited green spaces in low-income neighborhoods further contribute to the development of obesity among low SES populations in developed countries [70, 71]. The obesity–poverty paradox is yet another reminder of the importance of addressing obesity from multiple perspectives and accounting for geographic, social, and economic factors while designing and implementing obesity-targeted interventions.

#### 3.3.2. Neighborhood Characteristics

Neighborhood factors such as residential areas, neighborhood facilities, crime rate, and neighborhood type (rural vs. urban) play a crucial role in the development of childhood obesity [35]. Most studies focusing on neighborhood characteristics were conducted in North America (United States = 5, Canada = 1). The Canadian cross-sectional study focused on Aboriginal children (6–14 years), finding that living in a rural residential area was consistently associated with a higher likelihood of obesity among boys but not girls [30]. Similarly, studies in the United States found significant associations between neighborhood type, facilities, crime rate, and poverty. A cross-sectional study of fifth and sixth-grade students found elevated BMI significantly associated with increased property crime and living ≥ 0.5 miles from the nearest grocery store [34]. A similar longitudinal study among 0–17-year-olds also noted an increased risk of obesity associated with living in a neighborhood with no amenities [37]. Other US-based cohort studies had similar findings; children living in high-poverty areas with generally low education, high neighborhood disorganization, and a high density of fast food establishments had a higher risk of obesity compared to their peers.

Four other studies examined the impact of neighborhood characteristics on obesity. The first was an Iranian cross-sectional study that noted spatial trends in childhood obesity; the highest spatial risk was found among girls and boys residing in the north, northwest, and southwest regions of Iran (*p* < 0.05) [32]. Additionally, the risk was higher for those living in urban areas with extended exposure to smoke [32]. This finding was similar to a Ghanaian cross-sectional study that found a 32% higher likelihood of overweight and obesity among children (0–5 years) living in urban areas (PR = 1.32; 95% CI: 0.81, 2.18). A US-based prospective cohort study of American Indian and Native Alaskan children (2–11 years) also found a 28% higher odds of overweight and obesity for those living in counties with higher levels of poverty [36]. A similar trend was seen among native populations in Canada. Aboriginal boys residing in Atlantic provinces, Québec, British Columbia, and the Territories were less likely to be obese than boys living in Ontario. However, girls in the Atlantic provinces, Prairie regions, and the Territories were at higher risk than those in Ontario [30]. On the other hand, an Italian cross-sectional study had contradictory findings that children (5–11 years) living in rural areas had 2.55 times higher odds of obesity compared to those in urban areas (OR = 2.55; 95% CI: 1.03, 5.27) [33].

#### 3.3.3. Food Environment

Numerous studies have indicated a direct or indirect effect of food quality and access on obesity. However, studies examining this relationship among pediatric populations are limited. Most of these studies were conducted in the United States and Canada, with a few from Asian and European countries. A cross-sectional study of children (12–16 years) in California found that greater food insecurity was significantly associated with a greater BMI (*β* = 0.12, *p* = 0.015) and indirectly associated with waist circumference, with sleep health as a mediator [27]. This was substantiated by findings from another large US-based cross-sectional study [29]. Similar observations were made in a Canadian cross-sectional study, where off-reserve Indigenous children (6–17 years) and youth from households with very low food security were at a higher risk of overweight or obesity [28]. An Indonesian cross-sectional study also showed that food management, including total composition and type of food consumed, significantly predicted obesity among teenagers [45]. On the contrary, a few cross-sectional studies found no significant increase in the risk of obesity related to experiencing hunger due to food insecurity [30].

#### 3.3.4. Healthcare Access and Utilization

Access to affordable healthcare is an important determinant of health. This review identified one retrospective cohort study conducted in the United States that examined the impact of the type of health insurance coverage on childhood obesity. Notably, this retrospective chart review of EHRs found that publicly insured children (5–18 years) demonstrated a significantly greater increase in BMI *z*-score and weight percentile versus privately insured patients during the COVID-19 pandemic (*p* = 0.009) [24]. Other cross-sectional studies noted that lack of consistent health insurance was significantly associated with higher odds of overweight/obesity prevalence (aOR 1.3; 95% CI: 1.1, 1.7) [29], and having good health insurance coverage was protective against obesity [18].

#### 3.3.5. Immigration

Immigration is a significant SDOH that affects millions of individuals around the world. In the context of childhood obesity, two prospective cohort studies conducted in North America (Mexico and Canada) examined the impact of immigration and immigration networks on children's (5–15 years) metabolic health. The Canadian prospective cohort study found that immigrant children (3–13 years) had a significantly higher risk of having a waist circumference ≥ 90th percentile compared to refugee children (3–13 years), indicating a greater risk of overweight/obesity [26]. Furthermore, immigrant children (3–13 years) had significantly higher mean total body fat and mean trunk fat compared to refugee children, with those aged 8–13 being at higher risk of overweight based on their body fat percentage [26]. Similarly, the prospective cohort study in Mexico revealed that children (5–15 years) with connections to migrant networks had a greater risk of developing overweight or obesity relative to children without such network ties. This association was more pronounced among children in households with an extended family member in the United States [25]. Longitudinally, children (5–15 years) in Mexico who had migrant extended family networks in 2005 had an increased risk of overweight or obesity by 2009, indicating the long-term effects of immigration-related stressors on the children's metabolic health.

#### 3.3.6. Cultural and Structural Determinants

Complementary to immigration, other cultural and social factors are significant determinants of health and obesity among children. Social risk represents the collective impact of various SDOHs, including discrimination, social support, cohesion, and safety. A cross-sectional study based on the National Survey of Children's Health found that children (12–17 years) with low social support had higher odds of being overweight/obese (aOR: 1.3; 95% CI: 1.1–1.4), while those who experienced discrimination had an even higher risk (aOR: 1.4; 95% CI: 1.1, 1.8) [29]. These findings suggest that social support and lived experiences can have both direct and indirect effects on pediatric health.

Language spoken at home serves as a key cultural indicator. A Canadian cross-sectional study with Metis children found that those aged 6–10 years who spoke an Aboriginal language and those who spent time with elders ≥ 4 times/week had increased odds of obesity compared to those who did not. These associations differed by sex and age groups [30].

Additionally, there has also been a growing body of literature on the impact of religion on overall health. A cross-sectional study from Ghana indicated that children (0–5 years) whose heads of households did not follow a specific religion were twice as likely to be overweight or obese (PR = 2.02; 95% CI: 1.02, 4.03). This suggests that cultural and/or religious beliefs [41] may also influence a child's risk of developing overweight or obesity [41].

## 4. Discussion

This scoping review synthesized evidence on the SDOH associated with childhood obesity across different geographical regions. The findings underscore the multifaceted nature of childhood obesity, influenced by a combination of SES, neighborhood characteristics, food environment, healthcare access/utilization, immigration, and cultural factors.

Our review found a strong association between lower SES, characterized by lower family income, parental education, and employment status, and higher rates of childhood obesity [30, 43, 44, 48, 62, 63]. This is consistent with existing literature that indicates that lower SES often limits access to healthy food options, recreational facilities, and healthcare services [18, 24, 34, 37, 72], thereby increasing the risk of obesity. However, some studies from developing countries showed an opposite trend, with higher SES linked to obesity [49, 53, 54, 57, 58], potentially due to lifestyle changes associated with economic globalization [63].

The opposing trend in these countries can likely be attributed to several factors, primarily driven by economic globalization [73–75]. As countries experience rapid economic development, there are significant shifts in lifestyle, including dietary habits and physical activity patterns. Higher SES groups in developing countries often have increased access to Westernized food markets and processed foods, which are energy-dense but nutrient-poor [75]. This shift in diet, coupled with a decline in physical activity due to urbanization and sedentary lifestyles, can contribute to higher obesity rates. Additionally, higher SES groups in these countries may have more access to private transportation and leisure activities that promote sedentary behavior, further exacerbating the obesity epidemic. These factors highlight the complex relationship between SES and obesity in the context of economic development and cultural shifts.

The built environment significantly influences children's health behaviors. Our review found higher obesity rates among children in high-poverty areas with limited recreational facilities and green spaces. This aligns with previous literature suggesting that neighborhood disorganization, lack of recreational facilities, and high crime rates can restrict physical activity [76, 77]. Additionally, limited access to healthy and affordable food contributes to higher BMI and waist circumference among children, with food insecurity and poor dietary management exacerbating the issue [27–29]. These findings underscore the need for targeted interventions in disadvantaged neighborhoods that improve access to safe recreational spaces and healthy and affordable food options.

In addition to the built environment, limited access to healthcare services and inconsistent health insurance coverage were found to be associated with higher childhood obesity rates [18, 24], emphasizing the critical role of healthcare accessibility in managing and preventing obesity. Children with public insurance showed greater increases in BMI compared to those with private insurance, likely a reflection of racial and socioeconomic disparities in insurance type. Immigrant children, particularly those within migrant networks, were found to be at a higher risk of obesity [25, 26], with stress and challenges of immigration affecting dietary habits and physical activity. Cultural differences between their home and host countries, including native language and religious beliefs, further influence childhood obesity rates [78]. For instance, children who spoke an Aboriginal language or had no specific religious affiliations were found to have higher obesity rates, indicating the significant impact of cultural practices and beliefs on health behaviors [41]. Addressing these issues requires expanding healthcare coverage and improving accessibility to affordable services. Developing culturally sensitive health promotion programs that account for the unique needs of immigrant and culturally diverse children can also help mitigate obesity risks. Community-based interventions that promote healthy lifestyles and provide support systems for immigrants can foster environments that encourage healthier behaviors and reduce childhood obesity rates.

Despite extensive research on SDOH and childhood obesity, several gaps remain. A majority of relevant existing studies are cross-sectional and limited in their ability to understand temporal associations and long-term impacts. Further investigation into cultural and regional variations in the impact of SDOH on childhood obesity is also warranted to inform culturally tailored interventions and policies. Additionally, elucidating the underlying mechanisms through which various SDOH influence childhood obesity including biological, psychological, and social pathways needs to be further investigated.

Several limitations should be considered when interpreting the findings of this review. Our inclusion criteria limited this review to primary research articles available in English, potentially excluding relevant studies published in other languages. The exclusion of intervention studies, editorials, reports, and reviews may have led to the omission of valuable insights. Furthermore, the limited number of electronic databases used might have excluded pertinent studies not indexed in these databases or not available in an electronic format. The heterogeneity among included studies, varying widely in terms of geographic location, sample size, and study design, presents challenges with drawing definitive conclusions about social determinants of obesity among specific populations. Lastly, since most of the studies were cross-sectional, the ability to infer causality between various social and structural determinants and childhood obesity was limited.

## 5. Conclusion

This review highlights the significant role of SDOH in childhood obesity shaped by SES, neighborhood characteristics, food environment, healthcare access/utilization, immigration, and cultural factors. While lower SES is consistently linked to higher obesity rates, exceptions in some developing countries, potentially linked to globalization, demands further exploration. Effective public health interventions and policies require addressing these interconnected factors, prioritizing equitable access to healthy foods, safer neighborhood environments, and healthcare, especially in underserved communities. Culturally tailored, community-based programs are crucial, especially in diverse populations. Future research, including longitudinal studies, is needed to explore the long-term effects of SDOH on childhood obesity and health disparities, informing targeted and equitable public health strategies, promoting healthier futures for the children.

## Figures and Tables

**Table 1:** Characteristics of articles (*N* = 41) by social determinants of health domains, study location, children age categories, SDOH of interest, and weight-related outcome.

**Author(s) (year of publication)**	**Geographic locations**	**Children age categories**	**SDOH of interest**	**Outcome as defined**	**Outcome as measured**
*Healthcare access and utilization*					
Dopke et al. (2023) [24]	United States	5–8 years	• COVID-19 pandemic • Insurance type	General obesity	BMI *z*-score
*Immigration*					
Vilar-Compte et al. (2021) [25]	Mexico	5–15 years	• Migration	General obesity	BMI *z*-score
Lane et al. (2018) [26]	Canada	3–13 years	• Immigrant status	General obesity	BMI (kg/m^2^)
*Food insecurity*					
Dong et al. (2023) [27]	United States	12–16 years	• Food insecurity	General obesity, abdominal obesity	BMI *z*-score, WC
Bhawra et al. (2017) [28]	Canada	6–17 years	• Food security • Household characteristics • School environment	General obesity	BMI (kg/m^2^)
Brochier et al. (2023) [29]	United States	12–17 years	• Food insecurity • Discrimination, • Insurance coverage • Neighborhood cohesion • Neighborhood safety	General obesity	BMI (kg/m^2^)
*Neighborhood characteristics*					
Cooke et al. (2013) [30]	Canada	6–14 years	• Neighborhood factors • Socioeconomic factors	General obesity	BMI (kg/m^2^)
Baniissa et al. (2020) [31]	United Arab Emirates	13–19 years	• School neighborhood • School type	General obesity, abdominal obesity	BMI (kg/m^2^), WC, WHR, WHtR
Yazdi et al. (2020) [32]	Iran	7–19 years	• Geographic location • Urban vs. rural residence	General obesity, abdominal obesity	BMI (kg/m^2^) WC, WHtR
Protano et al. (2017) [33]	Italy	5–11 years	• Urbanization of residential area • Environmental tobacco smoke	General obesity	BMI (kg/m^2^)
Carroll-Scott et al. (2013) [34]	United States	10–12 years	• Neighborhood traits • Socioeconomic factors • Environmental factors	BMI	BMI (kg/m^2^)
Gupta et al. (2023) [35]	United States	0–7 years	• Neighborhood poverty • Crowded households • Single-parent households	General obesity	BMI percentile
Fyfe-Johnson et al. (2023) [36]	United States	2–11 years	• Urban–rural classification of residential area • Household income • Health insurance coverage, age, sex	General obesity	BMI percentile
Min et al. (2020) [37]	United States	10–18 years	• Neighborhood characteristics • Household income • Access to hospitals • Access to food and food expenditures • Number of grocery stores • Distance from home to grocery store	General obesity	BMI (kg/m^2^)
Yusuf et al. (2020) [18]	United States	0–17 years	• Poverty level • Number of neighborhood amenities • Household income	General obesity Overweight	BMI percentile
*Socioeconomic status*					
Cooke et al. (2013) [30]	Canada	6–14 years	• Household income • Parental education	General obesity	BMI (kg/m^2^)
Garibotti et al. (2015) [38]	Argentina	4–13 years	• Socioeconomic level • Maternal education • Adolescent pregnancy • Medical coverage • Neighborhood safety • Family habits	General obesity	BMI (kg/m^2^)
Rougeaux et al. (2017) [39]	United Kingdom	3–11 years	• Socioeconomic circumstances • Highest maternal academic attainment	Weight categories	BMI (kg/m^2^)
Moradi et al. (2017) [40]	Iran	10–12 years	• Socioeconomic inequalities	General obesity	BMI (kg/m^2^)
Atsu et al. (2017) [41]	Ghana	0–5 years	• Household data • Maternal education • Wealth index quintile	General obesity	Weight-for-height *z*-score (WHZ)
Donkor et al. (2017) [42]	Norway	5–6 years	• Early life SES predictors • Maternal age • Maternal education	General obesity Overweight	BMI (kg/m^2^)
Entezarmahdi et al. (2021) [43]	Iran	2–5 years	• Socioeconomic predictors	General obesity	BMI *z*-score
Djalalinia et al. (2021) [44]	Iran	6–18 years	• Socioeconomic inequality	General obesity Abdominal obesity	BMI (kg/m^2^) Waist circumference-to-height ratio
Nawangwulan et al. (2022) [45]	Indonesia	15–18 years	• Social and economic determinants	General obesity	BMI (kg/m^2^)
Balducci et al. (2022) [46]	Italy	8–9 years	• Economic difficulty • Parental education	General obesity	BMI (kg/m^2^)
Kim et al. (2023) [47]	Korea	12–18 years	• Socioeconomic inequalities	General obesity	BMI (kg/m^2^)
Buitrago-Lopez et al. (2015) [48]	Colombia	6–10 years	• Socioeconomic status	Overweight/obesity Abdominal obesity Insulin resistance	BMI *z*-score, WC, HOMA-IR
O'Dea et al. (2014) [49]	Australia	8–13 years	• Socioeconomic status	Overweight/obesity	BMI (kg/m^2^)
Assari et al. (2019) [50]	United States	12–17 years	• Parental education attainment	General obesity	BMI (kg/m^2^)
Kain et al. (2019) [51]	Chile	4–6 years	• Attending a very vulnerable school • Being indigenous • Maternal education • Being cared for by the grandmother after school	General obesity	BMI *z*-score
Kim et al. (2022) [52]	United States	2–11 years	• Race/ethnicity • Socioeconomic status	General obesity	BMI *z*-score
Noonan et al. (2018) [53]	United Kingdom	3–14 years	• Family income • Poverty	Weight categories	BMI (kg/m^2^)
Lai et al. (2023) [54]	United Kingdom	9 months–14 years	• Poverty dynamic	General obesity	BMI (kg/m^2^)
Stangvaltaite-Mouhat et al. (2021) [55]	Norway	15–19 years	• Adolescents program of study • Parental education • Parent employment	Overweight/obesity	BMI (kg/m^2^), WC
Hazrati et al. (2019) [56]	United States	1 year	• Maternal ethnicity • Maternal education • Maternal stress	Weight gain	Weight-for-length (WFL) percentiles
Wijesundera et al. (2023) [20]	Canada	4–6 years	• Material and social depravation • Maternal immigration status	General obesity	BMI *z*-score
Cook et al. (2016) [57]	United States	12–17 years	• Family income • Parent education • Nativity status	General obesity	BMI percentile
Conrey et al. (2022) [58]	United States	0–2 years	• Poverty • Depravation • Maternal education • Marital status • Insurance status	General obesity	BMI *z*-score
Tester et al. (2018) [59]	United States	2–5 years	• Sociodemographics	Severe obesity	BMI percentile
Zhuang et al. (2022) [60]	United States	5–18 years	• Time-varying maternal precarious employment	General obesity	BMI percentile
Vale et al. (2022) [61]	Brazil	10–19 years	• Number of people in household • Public vs. private schools • Geographic region • Fast food intake	General obesity Stunting	BMI *z*-score, BMI (kg/m^2^), height measurement

*Note:* HOMA-IR = homeostatic model assessment of *β* cell function and insulin resistance.

Abbreviations: BMI, body mass index; WHR, waist-to-hip ratio; WtHR, waist-to-height ratio.

**Table 2:** Characteristics of articles (*N* = 41) by social determinants of health domains, study location, years of data collection, analysis, and weight-related outcome.

**Author(s) (year of publication)**	**Study design**	**Year of data collection**	**Data collection and analysis**	**Results (main findings)**
*Healthcare access and utilization*				
Dopke et al. (2023) [24]	Retrospective cohort	2018–2020	• Medical charts • Linear regression models	• Publicly insured patients demonstrated significantly greater increase in BMI *z*-score vs. privately insured patients (*p* = 0.009). • Mean differences between groups increased from 0.26 in 2018 (95% CI [0.07, 0.45]) to 0.42 in 2020 (95% CI [0.23, 0.61]).
*Immigration*				
Vilar-Compte et al. (2021) [25]	Prospective cohort	2005 and 2009	• Mexican Family Life Survey • Two-level random-intercept logistic models	• Children embedded in migrant networks are at a greater risk of developing overweight or obesity relative to children with no network ties to the United States. • This association is larger and more significant among children in households with an extended family member in the United States.
Lane et al. (2018) [26]	Prospective cohort	2011 and 2012	• Interviews, questionnaires, and physical examination • Univariate/multivariate logistic regression	• Older children, those with better-educated parents, and those who consumed a poorer-quality diet were at a higher risk of being overweight or obese. • Compared with refugee children, immigrant children aged 11–13 years were at a significantly higher risk of having waist circumference ≥ 90th percentile.
*Food insecurity*				
Dong et al. (2023) [27]	Cross-sectional	2018–2020	• Home visits with surveys, anthropometric assessments, blood draws, 7-day actigraphy with sleep diaries • Multiple regression analysis	• Greater food insecurity was significantly associated with greater BMI (*b* = 0.12, *p* = 0.015). • There was a significant indirect path from greater food insecurity to greater waist circumference through poorer sleep health (0.11, 95% bootstrapping CI: [0.01, 0.30]).
Bhawra et al. (2017) [28]	Cross-sectional	2012	• 2012 Aboriginal Peoples Survey • Pearson chi-squared test and multivariable logistic regression models	• Off-reserve Indigenous children and youth from households with very low food security were at higher risk of overweight or obese status. • Negative school environment was also a significant predictor of obesity risk.
Brochier et al. (2023) [29]	Cross-sectional	2017 and 2018	• National Survey of Children's Health • Multivariable logistic regression models	• Food insecurity, caregiver underemployment, low social support, and discrimination were significantly associated with higher obesity prevalence. • For each additional social risk factor a child was exposed to, their odds of having overweight/obesity were aOR: 1.2, 95% CI: [1.2, 1.3].
*Neighborhood characteristics*				
Cooke et al. (2013) [30]	Cross-sectional	2006	• Questionnaire • Logistic regression	• The effects of socioeconomic factors and region varied across age and gender groups, although living in a lone-parent family and rural residence had consistent effects. • PMK residential schooling was positively associated with obesity generally.
Baniissa et al. (2020) [31]	Cross-sectional	2018 to 2019	• Physical Activity Questionnaire for Adolescents, demographic questionnaire, and National Cancer Institute (NCI) Fruit and Vegetable Screener • Chi-square tests, mixed effect analysis, and multivariable logistic regression model	• More participants from public schools were overweight/obese (37.8% vs. 31.1%) and had greater abdominal obesity compared with those from private schools. • Predictors of abdominal obesity were studying at public school (WHR: AOR 1.67, 95% CI: 1.06–2.66) and being Emirati (WHR: aOR 0.62, 95% CI: 0.43–0.90).
Yazdi et al. (2020) [32]	Cross-sectional	2011–2012	• WHO-GSHS questionnaire and anthropometric measurements • Bayesian spatial modeling, C2 test, and MCMC algorithm	• Risk of obesity was significantly higher in areas with a higher rate of urban vs. rural residence. • The highest spatial risks of general obesity and abdominal obesity were observed in the north, northwest, and southwest of Iran.
Protano et al. (2017) [33]	Cross-sectional	2014	• Questionnaires and face-to-face interviews • Chi-square tests, multivariate logistic regression, and backward stepwise selection	• Living in a rural area (adjusted OR, 2.55; 95% CI: 1.18–5.52) and lower maternal education (adjusted OR, 2.32; 95% CI: 1.03–5.27) were significant predictors of overweight/obese status.
Carroll-Scott et al. (2013) [34]	Cross-sectional	2009	• Anthropometry, school district data systems, and US census • Linear regression models and backward stepwise model selection	• Higher BMI was significantly associated with living more than a half mile from the nearest grocery store and living in neighborhoods with more property crimes.
Gupta et al. (2023) [35]	Prospective cohort	2002–2019	• US census data and health records • Latent class growth modeling, multinomial regression analysis, and heat mapping	• Children at high risk of obesity were from neighborhoods with more crowded households (*p* < 0.001), lower socioeconomic status (*p* < 0.001), higher minority population (*p* < 0.05), single-parent households (*p* < 0.001), lower educational status with high high-school dropout (*p* < 0.05) and low preschool enrolment (*p* < 0.05), and greater rurality (*p* < 0.001).
Fyfe-Johnson et al. (2023) [36]	Prospective cohort	2010–2014	• Indian Health Services databases, American Community Survey, Census Bureau, USDA, Environment Atlas, and National Center for Health Statistics • Multiple GLM models	• Children living in counties with higher levels of poverty had 28% higher odds of prevalent overweight/obesity status. • Children living in counties with more children eligible for free or reduced-priced lunch had 15% lower odds for transitioning from normal weight status to overweight/obesity status.
Min et al. (2020) [37]	Prospective cohort	2011–2018	• Anthropometry and US census bureau • Chi-square tests, *t*-tests, nonparametric tests, linear and logistic regression models, and hot spot analysis	• Obese patients were more likely to reside in a disadvantaged neighborhood, with higher access corner stores (*p* < 0.001) and marginally higher ratio in fast food to total food expenditure (*p* = 0.05) than nonobese patients. • The level of BMI percentile significantly increased with neighborhood disorganization level.
Yusuf et al. (2020) [18]	Cross-sectional	2016–2017	• National Survey of Children's Health 2016–2017 • Pearson chi-square test, log binomial logistic regression models, and prevalence ratios	• Overweight was more frequent in younger children, children of single parents, and children who lived in a neighborhood with no amenities. • Parental attainment of college education, health insurance coverage, female gender, and language spoken in home other than Spanish were protective against overweight or obesity.
*Socioeconomic status*				
Cooke et al. (2013) [30]	Cross-sectional	2006	• Questionnaire • Logistic regression	• The effects of socioeconomic factors and region varied across age and gender groups, although living in a lone-parent family and rural residence had consistent effects. • PMK residential schooling was positively associated with obesity generally.
Garibotti et al. (2015) [38]	Cross-sectional	2008–2009	• Surveys, anthropometric measurement, and oral examination • Pearson chi-square test and logistic regression model	• No relationship between weight/obesity and studied sociodemographic characteristics was observed.
Rougeaux et al. (2017) [39]	Prospective cohort	2000–2002	• Interviews, anthropometric measurements, Strengths and Difficulties Questionnaire (SDQ) • GEE models, Poisson regression analysis, sensitivity analyses, and imputations	• By age 11, children with mothers who had no academic qualifications were considerably more likely to be overweight compared with those with degree-educated mothers (PR = 1.6 (95% CI 1.4–1.8), PD = 12.9% (9.1%–16.8%)).
Moradi et al. (2017) [40]	Cross-sectional	2015	• Survey, anthropometric measurement • Principal component analysis (PCA), Oaxaca decomposition, and logistic regression analyses	• The concentration index for overweight and obesity, respectively, was 0.10 (95% CI: 0.05, 0.15) and 0.07 (95% CI: 0.00, 0.14) which indicated inequality and a higher prevalence of obesity and overweight in higher SES.
Atsu et al. (2017) [41]	Cross-sectional	2010–2021	• 2-stage survey, physical examinations • Bivariate Poisson regression and multivariate Poisson regression	• Children whose household heads did not belong to any religion had more than twice the rates of obesity than those from the Christian religion (PR = 2.024, 95% CI, 1.016, 4.034). • Children belonging to the fourth index quintile vs. the poorest quintile had a significantly increased rates of overweight (aPR = 1.010; 95% CI, 1.010–1.017). • Children from the rural vs. urban areas were more likely to be overweight (PR = 1.329; 95% CI, 0.810–2.183).
Donkor et al. (2017) [42]	Prospective cohort	2007	• Anthropometric measurements, parental questionnaire • ANOVA, chi-square tests, ordinal bivariate logistic regression analyses, and multinomial logistic regression analysis	• High BMI at 5 years of age was related to a wide range of unfavorable sociodemographic and behavioral factors (i.e., high parental BMI).
Entezarmahdi et al. (2021) [43]	Cross-sectional	2017	• Interviews, questionnaire, and anthropometric measurement • Logistic regression	• The likelihood of obesity among children with high and moderate SES was 2.6 and 1.6 times more compared to children with low SES, respectively.
Djalalinia et al. (2021) [44]	Cross-sectional	2011	• Student and parent questionnaires, interviews, and semiquantitative food frequency questionnaires • Multiple logistic regression analysis, *C* index, linear regression analysis, and Blinder–Oaxaca decomposition methods	• The prevalence of excess weight, overweight, and general and abdominal obesity increased linearly as SES increased. • Regarding overweight, excess weight, general and abdominal obesity, the index was positive, indicating that inequality was in favor of in low SES groups.
Nawangwulan et al. (2022) [45]	Cross-sectional	2020–2021	• Interviews and observations • Chi-squared tests, univariate/bivariate logistic analysis, and multivariate logistic regression analysis	• The education level of mother (*p* = 0.364) and father (*p* = 0.142) did not significantly influence obesity in teenagers. • Parents' income did not significantly influence the obesity of teenagers. • Food management was significant (*p* = 0.002) and influenced obesity in teenagers.
Balducci et al. (2022) [46]	Cross-sectional	2019	• Anthropometric measurements and surveys • Prevalence estimates, prevalence ratios, and chi-square tests	• 43% of obese Apulian children who have parents with low levels of education and severe economic difficulties would be able to reduce their weight status if social inequalities were changed. • Obesity in children is closely related to the socioeconomic status of the parents: The higher the level of income inequality, the more the children are overweight.
Kim et al. (2023) [47]	Cross-sectional	2006–2020	• The Korea Youth Risk Behavior Web-based Survey • Trend analyses, prevalence ratio, prevalence difference, and log-binomial regression models	• A greater disparity in obesity was observed according to the paternal education 2.18 (95% CI, 2.14–2.22), maternal education 1.75 (95% CI, 1.72–1.78), and household economic status 1.50 (95% CI, 1.48–1.52). • Girls showed higher values: 2.49 (95% CI, 2.42–2.55) for income, 3.17 (95% CI, 3.08–3.26) for paternal education, and 2.62 (95% CI, 2.55–2.70) for maternal education compared to boys.
Buitrago-Lopez et al. (2015) [48]	Cross-sectional	2006–2007	• Anthropometric measurements and clinical visits • Logistic regression	• Children in the highest SES had higher odds of overweight/obesity (OR = 3.25, 95% CI 1.89–5.57) and abdominal obesity (OR = 2.74, 95% CI 1.41–5.31).
O'Dea et al. (2014) [49]	Prospective cohort	2007	• Anthropometric measurements • Chi-square tests and linear mixed models	• Males and females in the low SES group were more likely to be obese (6%–7%) than middle (4%–5%) and high (2%–3%) SES groups.
Assari et al. (2019) [50]	Cross-sectional	2013–2014	• Questionnaire • Linear regression models	• Higher parental educational attainment was associated with lower youth BMI. • Race moderated the effect of parental educational attainment on BMI, suggesting that the protective effect of parental educational attainment on BMI is significantly smaller for black than white youth.
Kain et al. (2019) [51]	Prospective cohort	2011–2015	• Surveys • BMI *z*-scores for weight categories, logistic regression analysis, and stratified analysis by sex	• Excessive weight gain was associated with the child looked after school by a grandmother, attending a very vulnerable school, being indigenous, and mother having only primary education.
Kim et al. (2022) [52]	Prospective cohort	2013–2015	• Medical charts/records and interviews • Maximum likelihood estimation, Bayesian information criterion (BIC), and ordinal logistic regression analyses	• Children whose parents had a college or postcollege education had lower odds of being in the upper trajectory vs. the mid/lower trajectory compared to children whose parents had less than a high school education (reference group). • Children whose family income level was greater than $100,000 had significantly lower odds of obesity compared to those whose family income was less than $20,000 (reference group).
Noonan (2018) [53]	Cross-sectional	2000–2002	• Precollected by MCS study and questionnaire • Logistic regression analysis	• Boys (OR = 1.39, 2.04; *p* < 0.001) and girls living in poverty (OR = 1.55, 2.24; *p* < 0.001) were more likely to be classified as overweight and obese compared to those not living in poverty. • The lowest income adolescents were more likely to be overweight/obese compared to the second to least (OR = 1.18, 1.27; *p* < 0.05), third to least (OR = 1.34, 1.81; *p* < 0.001), fourth to least (OR = 1.64, 2.33; *p* < 0.001), and highest income adolescents (OR = 2.10, 4.11; *p* < 0.001).
Lai et al. (2023) [54]	Prospective cohort	2000–2002	• Demographics collected by MCS study, Strengths and Difficulties Questionnaire (SDQ), and anthropometric measurements • Pearson chi-square test and multivariable logistic regression	• Persistent poverty, when compared with those never in poverty, was associated with higher risk of being obese (OR: 2.21; 95% CI: 1.80–2.72) at age 14 years. • The associations of predicted poverty trajectories and child's health outcomes did not vary by sex since (*p* > 0.05).
Stangvaltaite-Mouhat et al. (2021) [55]	Cross-sectional	2010–2011	• Anthropometric measurements, physical examination, and e-questionnaire • Multivariable logistic regression analyses	• Boys enrolled in the general studies (vs. vocational) had lower odds of being overweight/obese (POR 0.42, 95% CI 0.20–0.86), of having high waist circumference (POR 0.39, 95% CI 0.21–0.75 and POR 0.25, 95% CI 0.10–0.64, respectively).
Hazrati et al. (2019) [56]	Prospective cohort	2012	• Questionnaire, medical records • Chi-square test, multiple logistic regression models, forest plots, and k-means clustering technique	• Excess weight for Hispanic children was associated with lower maternal education (OR 2.37 (1.1–4.5)).
Wijesundera et al. (2023) [20]	Retrospective cohort	2009–2017	• Immunization visits and Alberta Vital Statistics Birth Registry • Multinomial logistic regressions and generalized estimating equations	• Children with maternal immigrant status (RRR = 0.71, 0.66–0.77) and every CAD 10,000 increase in income (RRR = 0.88, 0.86–0.90) were less likely to have obesity. • Children in the most materially deprived quintile were more likely to have overweight (RRR = 1.52, 1.46–1.58) and obesity (RRR = 2.83, 2.54–3.15).
Cook et al. (2016) [57]	Cross-sectional	2007–2012	• Questionnaire (CHIS), random digit dialing • Univariate analysis, bivariate analysis, multivariable logistic regression	• Having a relatively low family income of less than 300% of the family poverty line was significantly associated with higher odds of being overweight (OR = 2.34). • Parental education level was not significantly associated with odds of being overweight. Males (OR = 3.20) were over 3 times as likely to be overweight as females. • Neither US nativity nor lifestyle factors such as fast food consumption or physical inactivity were significantly associated with overweight in Asian American adolescents.
Conrey et al. (2022) [58]	Prospective cohort	2017–2020	• Precollected by PREVAIL • Spearman correlations, ANOVA, univariable analysis, multivariable regression analysis, and multivariable GEE model	• Residing outside of the highest SEE neighborhoods was associated with an increased BMIz of 0.04 (95% CI 0.02, 0.06) per month of life and increased obesity risk at age two (aRR: 3.7, 95% CI 1.2, 16.2), controlling for family sociodemographics.
Tester et al. (2018) [59]	Cross-sectional	1999–2014	• Questionnaire and anthropometric measurements • Linear regression and log-transformation	• Children with severe obesity had higher (unadjusted) odds of being from households with lower educational attainment (OR: 2.4), that were single parent headed (OR: 2.0), and that were in poverty (OR: 2.1).
Zhuang et al. (2022) [60]	Prospective cohort	1996–2016	• National Longitudinal Survey of Youth • Multidimensional precarious employment score (PES), linear generalized estimating equation models, Kaplan–Meier curves, and Poisson regression model	• Higher maternal precarious employment was associated with a 10% higher incidence of children having overweight/obesity (CI: 1.05, 1.14).
Vale et al. (2022) [61]	Cross-sectional	2015	• 2015 National School Health Survey • Pearson chi-square test, multilevel poison techniques, and prevalence ratios	• The prevalence of obesity among Brazilian adolescents (10.0%; 95% CI: 9.4–10.6) was associated directly with indifference or dissatisfaction with body image, with living with up to four people in the household, studying in private schools, and being from the South region and was inversely associated with being female, 15 years old or older.

*Note:* MCMC algorithm, Markov chain Monte Carlo; PMK, person most knowledgeable about the child; WHO-GSHS, World Health Organization Global School–based Student Health Survey; PREVAIL, Pediatric Respiratory and Enteric Virus Acquisition and Immunogenesis Longitudinal Cohort.

Abbreviations: ANOVA, analysis of variance; aRR, adjusted risk ratio; BMI, body mass index; CAD, Canadian dollar; CHIS, California Health Interview Survey; CI, confidence intervals; GEE, generalized estimating equation; MCS, Millennium Cohort Study; OR, odds ratio; POR, prevalence odds ratio; PR, prevalence ratio; RRR, relative risk ratio; SES, socioeconomic status; US, United States; WHR, waist-to-hip ratio.

## Data Availability

The list of articles included in this review are available from the corresponding author upon reasonable request.

## Nomenclature

SDOH: social determinants of health
WHO: World Health Organization
SES: socioeconomic status
US: United States
BMI: body mass index
CI: confidence interval
PR: prevalence ratio
OR: odds ratio
EHR: electronic health records
aOR: adjusted odds ratio
